# *Nepenthes* pitchers are CO_2_-enriched cavities, emit CO_2_ to attract preys

**DOI:** 10.1038/s41598-017-11414-7

**Published:** 2017-09-12

**Authors:** Sabulal Baby, Anil John Johnson, Elavinamannil Jacob Zachariah, Abdul Azeez Hussain

**Affiliations:** 1 Phytochemistry and Phytopharmacology Division, Jawaharlal Nehru Tropical Botanic Garden and Research Institute, Pacha-Palode, Thiruvananthapuram, 695 562 Kerala India; 2 Atmospheric Sciences Division, National Centre for Earth Science Studies, Post Box No. 7250, Akkulam, Thiruvananthapuram, 695 011 Kerala India; 3 Garden Management Division, Jawaharlal Nehru Tropical Botanic Garden and Research Institute, Pacha-Palode, Thiruvananthapuram, 695 562 Kerala India

## Abstract

Carnivorous plants of the genus *Nepenthes* supplement their nutrient deficiency by capturing arthropods or by mutualistic interactions, through their leaf-evolved biological traps (pitchers). Though there are numerous studies on these traps, mostly on their prey capture mechanisms, the gas composition inside them remains unknown. Here we show that, *Nepenthes* unopened pitchers are CO_2_-enriched ‘cavities’, when open they emit CO_2_, and the CO_2_ gradient around open pitchers acts as a cue attracting preys towards them. CO_2_ contents in near mature, unopened *Nepenthes* pitchers were in the range 2500–5000 ppm. Gas collected from inside open *N. khasiana* pitchers showed CO_2_ at 476.75 ± 59.83 ppm. CO_2_-enriched air-streaming through *N. khasiana* pitchers (at 619.83 ± 4.53 ppm) attracted (captured) substantially higher number of aerial preys compared to air-streamed pitchers (CO_2_ at 412.76 ± 4.51 ppm). High levels of CO_2_ dissolved in acidic *Nepenthes* pitcher fluids were also detected. We demonstrate respiration as the source of elevated CO_2_ within *Nepenthes* pitchers. Most unique features of *Nepenthes* pitchers, viz., high growth rate, enhanced carbohydrate levels, declined protein levels, low photosynthetic capacity, high respiration rate and evolved stomata, are influenced by the CO_2_-enriched environment within them.

## Introduction

*Nepenthes* consists of approx. 160 currently described species distributed in the Madagascar-south east Asia-north Australia-New Guinea region, with hotspots in Borneo, Sumatra and the Philippines. They grow in wet, sunny and nutrient (N, P)-poor habitats. In order to supplement this nutrient deficiency, they evolved strategies to capture insects and other arthropods through their modified leaf tips (pitchers or pitfall traps)^1–8^. The known factors attracting arthropod preys into the ‘passive’ *Nepenthes* traps are nectar, olfactory cues, colour and UV/fluorescence patterns^1, 3, 6^. Toxic metabolites, waxes, physical phenomena, viscoelastic pitcher fluid, chitinases/proteases and antifungal metabolites are also involved in various stages of carnivory displayed by these unique plants^2, 4, 5, 7^. Other than ‘arthropod trapping strategies’, recent reports show that, pitchers of Bornean *Nepenthes* species display ‘mutualistic interactions’ with tree shrews, bats and other small mammals, and thereby gain nutrients^8^.

*Nepenthes* leaves are highly specialized with two distinct portions, lamina and the pitcher (prey trap). The midribs of *Nepenthes* leaves protrude from the leaf tip into tendrils, form small buds which inflate into bulb- or tube-shaped pitchers. In other words, *Nepenthes* pitchers are modified epiascidiate leaves in which their adaxial (upper) surface curls around and fuses to form the inner side of the pitcher^1^. The tendrils of aerial pitchers are usually coiled in the middle, and once in contact with other objects for long enough they curl around them, forming anchor points for pitchers. In this way, *Nepenthes* tendrils help to support the growing stem of the plant. As it matures, the pitcher inflates and gets partially filled with an acidic enzymatic fluid. Pitchers also have a flap (operculum), which initially seals (‘hermetically seals’) the growing trap^1^, and once mature breaks open for prey capture. In *N. khasiana*, initial development stages to lid opening of pitchers take about 3 weeks. *N. khasiana* pitchers grow up to an average of 13 cm length, with lid length 3 cm and pitcher fluid 3.25 mL. In most *Nepenthes* species, the lid covers the pitcher opening and thus protects it from rain, preventing dilution of the pitcher fluid, but in some species the lids are reduced or bent backwards^9^. Once open, pitcher rims (peristomes) play major initial steps in attracting and capturing preys^2^. *Nepenthes* species show considerable variations in size, shape and colour of their pitchers (Fig. S1) and peristomes (Figs S2–S4). *N. rajah*, largest pitcher/carnivorous plant, grows up to 3 m in height, its pitchers grow up to 30 × 14 cm (height or length x width) and secrete up to 2.5 liters of pitcher fluid. The recently described species, *N. attenboroughii* and *N. palawanensis*, also produce large pitchers^10^. Large *Nepenthes* pitchers are capable of trapping rodents, lizards and birds. Once open, *Nepenthes* pitchers involve in prey capture from a few weeks up to nine months depending on the species^11^.

*Nepenthes* pitchers are even described as ‘hollow leaves’ in the literature^12^, but they are not entirely ‘hollow’. Unopened *N. khasiana* pitchers, on pressing with our hands, give a gas-filled sensation, and on further forcing they burst open mostly at the peristome-lid portion. *Nepenthes* pitchers, their growth, morphology, prey capture, mutualistic interactions, digestion mechanisms and nutrient uptake received lot of attention in recent decades^2, 4, 8, 13^. But, the gas composition inside *Nepenthes* pitchers has not been studied so far. In our preliminary tests, we found high levels of CO_2_ inside growing, unopened *N. khasiana* pitchers. This led us to look into the role of this CO_2_ in prey capture, growth and other unique features of *Nepenthes* pitchers.

## Results

### CO_2_ in *Nepenthes* pitchers, prey capture

We found growing, unopened pitchers of *N. khasiana* (Fig. S1a) filled with high levels of CO_2_ (4053.76 ± 1188.84 ppm, n = 9), along with ambient levels of O_2_, CO, CH_4_ and N_2_O. Various *Nepenthes* hybrids also showed high contents of CO_2_ in their growing (unopened) pitchers (Fig. S1b–g) (*Nepenthes* hybrid 01, NH01 3114.38 ± 973.52 ppm, n = 5; NH02 4008.67 ± 1042.38 ppm, n = 3; NH03 3390.03 ppm, n = 1). Gas samples from inside (just below the peristomes) open *N. khasiana* pitchers showed CO_2_ levels at 476.75 ± 59.83 ppm (n = 6). Moreover, open *N. khasiana* pitchers when their lids sealed back (after 24 hours of lid opening) regained the high CO_2_ levels (3231.33 ± 762.58 ppm, n = 3). Mature, unopened pitchers when cut open and sealed again (after 24 hours) also showed high contents of CO_2_ inside them (3324.00 ± 959.23 ppm, n = 3). Ambient CO_2_ levels at the *Nepenthes* experimental fields were 396.97 ± 6.07 ppm, n = 3, matching global measurements.

Near mature, unopened *N. khasiana* pitchers, when cut open and quickly re-weighed, showed noticeable reduction in their weights (*N. khasiana* pitcher length 12.94 ± 3.11 cm, lid length 3.01 ± 0.79 cm, pitcher fluid 3.25 ± 2.29 mL, weight difference 2.50 ± 1.58 mg, n = 45) (Table S1). *N. khasiana* individual pitchers showed weight differences from 0.80 to 8.50 mg (one exceptionally big pitcher) (Table S1). *Nepenthes* hybrid pitchers also showed similar weight reduction viz., 0.70 mg (NH 05) to 5.50 mg (NH 01) (details in Table S1).

**Figure 1:**
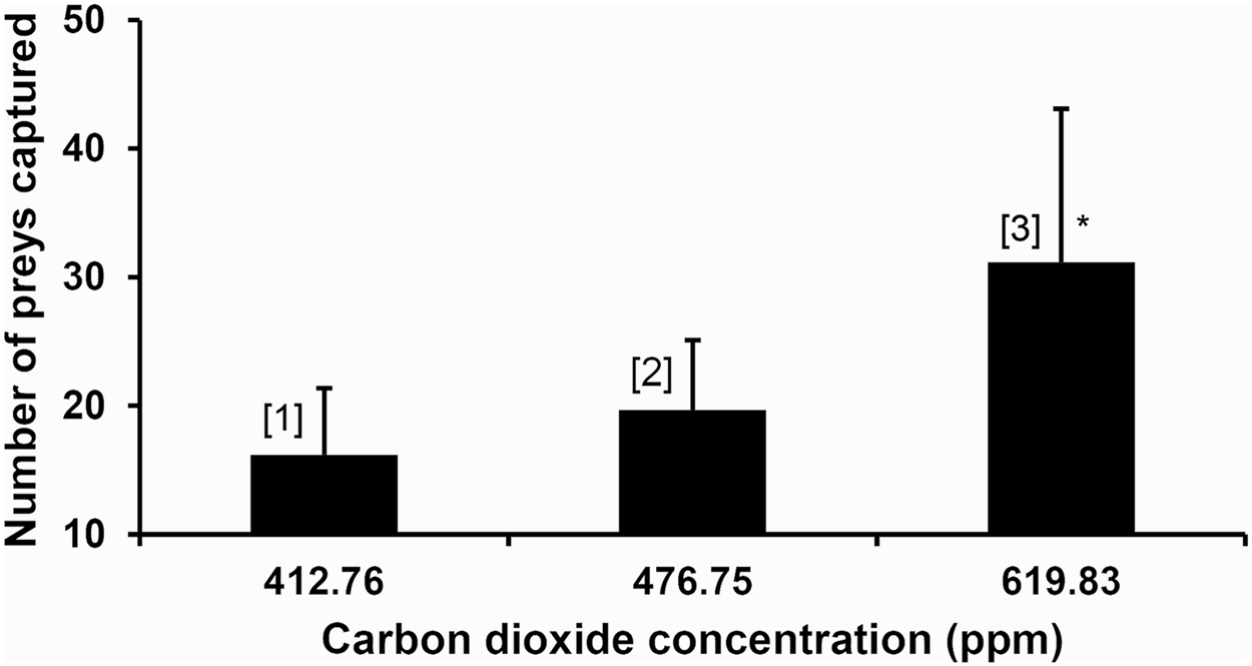
Prey capture rates enhance on streaming CO_2_ through *Nepenthes* pitchers. Preys captured in 12 days by [1]: air-streamed (control) *N. khasiana* pitchers (mean ± s.d., n = 6); [2]: normal (unmodified) *N. khasiana* pitchers (mean ± s.d., n = 6); [3]: CO_2_-streamed (test) *N. khasiana* pitchers (mean ± s.d., n = 6; ^*^significant at *p* < 0.05, compared to [2]).

We passed a stream of CO_2_-enriched air (1% CO_2_ in air) through the upper portion (above the liquid zone) of just opened *N. khasiana* pitchers in the field for 12 days. This CO_2_-enriched air, mixed with the gas inside the pitcher and discharged through the top of open pitchers (CO_2_ at 619.83 ± 4.53 ppm, n = 6), attracted substantially higher number of aerial preys (insects) (31.17 ± 11.91, n = 6, Fig. 1) into these traps. In control experiments, when a stream of air at the same flow rate was passed through *N. khasiana* pitchers (CO_2_ at 412.76 ± 4.51 ppm, n = 6) for 12 days, we found a relatively lower rate of insect capture, 16.2 ± 5.15 (n = 6). Capture rate in normal (unmodified) pitchers (CO_2_ at 476.75 ± 59.83 ppm, n = 6) for 12 days was 19.67 ± 5.43 (n = 6) (Fig. 1).

### CO_2_ in *Nepenthes* pitcher fluid

**Figure 2:**
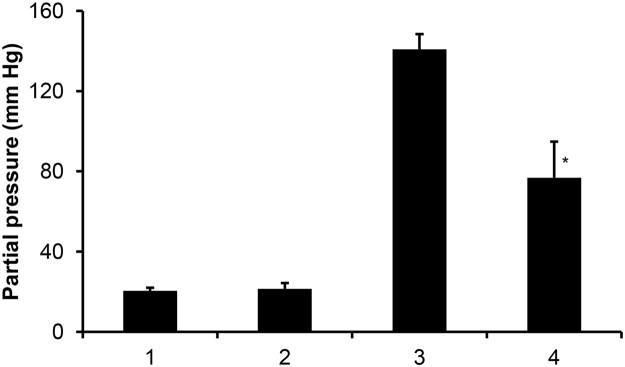
Partial pressures of CO_2_ (*p*CO_2_) and oxygen (*p*O_2_) in mature, unopened and opened, prey captured *N. khasiana* pitcher fluids. *p*CO_2_ in (1) mature, unopened and (2) opened, prey captured pitcher fluids (mean ± s.d., n = 6); *p*O_2_ in (3) mature, unopened and (4) opened, prey captured pitcher fluids, respectively (mean ± s.d., n = 6; ^*^significant at *p* < 0.05, compared to (3)).

On the average, *N. khasiana* pitchers produce pitcher fluids at 3.25 ± 2.29 mL (n = 45) (Table S1). Our data show that closed (unopened) *N. khasiana* pitchers have CO_2_-enriched gaseous media above their aqueous pitcher fluids, and CO_2_ remains in equilibrium with these fluids. Partial pressures of oxygen (*p*O_2_) and CO_2_ (*p*CO_2_) in mature, unopened *N. khasiana* pitcher fluids were measured as 140.83 ± 7.60 (n = 6) and 20.47 ± 1.53 mm Hg (n = 6), whereas *p*O_2_, *p*CO_2_ for opened, prey captured pitcher fluids were 76.78 ± 18.10 (n = 6) and 21.43 ± 2.85 mm Hg (n = 6), respectively (Fig. 2). *p*O_2_ and *p*CO_2_ in the atmosphere are 159 and 0.30 mm Hg, respectively. We also detected CO_2_ dissolved in *N. khasiana* pitcher fluid by headspace GC-MS (VF-5 column, ret. time 1.65 min; EI-MS, *m/z*: 44 (M^+^), 32). Mass data of CO_2_ from the pitcher fluid matched with its authentic standard. We measured the pH of unopened *N. khasiana* pitcher fluid as 3.54 ± 0.09 (n = 4), and on prey capture the fluid became more acidic (pH 2.47 ± 0.25, n = 4).

### CO_2_, lid opening, chemical defense

We observed prey captured pitcher fluids in open *N. khasiana* pitchers turning yellow whereas fluids in netted, open pitchers (with no ants or insects captured) remained colourless. DART-MS of yellow pitcher fluids showed droserone (MW 204.18) and 5-O-methyl droserone (MW 219.00) in them (Figs S5–S7). Chitin induction, mimicking prey capture, into *N. khasiana* pitcher fluid also turned it yellow and demonstrated the release of these antifungal metabolites in DART-MS^5^ (Figs S5–S7).

### CO_2_, stomata in *Nepenthes* pitchers

**Figure 3:**
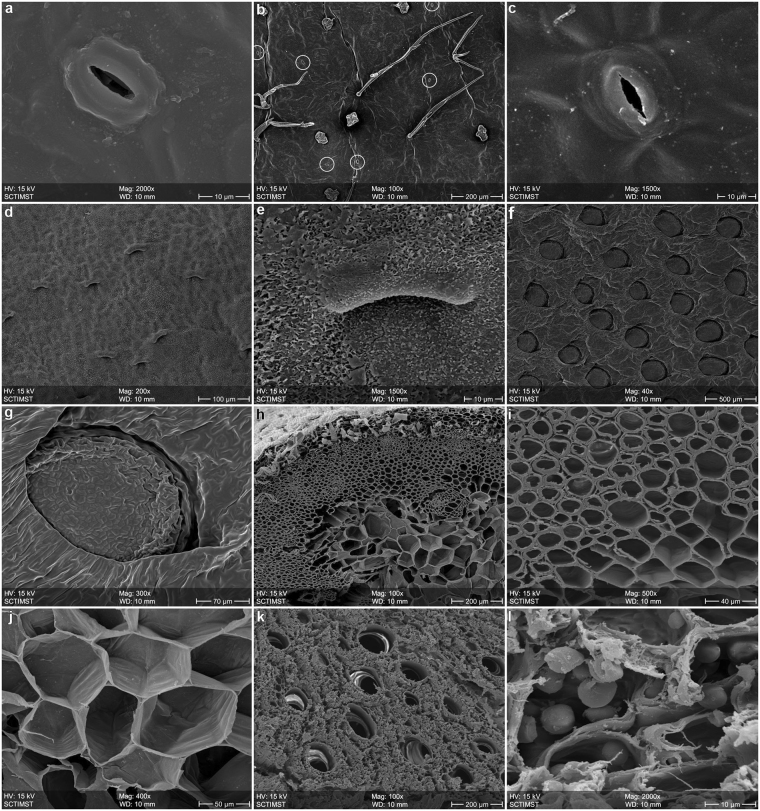
SEM images of root, leaf, tendril and pitcher of *N. khasiana*. (**a**), Stoma at the leaf abaxial (lower) surface. (**b**) Outside surface of pitcher with low stomatal density (stomata circled). (**c**) Stoma at the outside surface of pitcher. (**d**) ‘Stomatal’ distribution at the inner surface pitcher. (**e**) ‘Modified stoma’ at the inner pitcher surface, embedded in wax crystals. (**f**) Inner liquid zone of the pitcher showing glands. (**g**) Expansion of a secretory gland. (**h**) Tendril cross section, showing vascular openings. (**i**) Tendril cross section, outer layer. (**j**) Tendril cross section, central portion. (**k**) Root cross section with vascular openings. (**l**) Root cross section showing starch granules.

In SEM images, we found *N. khasiana* leaves (laminae) hypostomatic i.e., stomata observed only in their abaxial (lower) sides (Fig. 3a), and not in adaxial (upper) sides. But *N. khasiana* pitchers (both unopened and open pitchers) showed stomata in their outer sides, and ‘modified stomata’ in their inner sides (Fig. 3b–e). No stomata were seen at the inner sides of *N. khasiana* pitcher lids (Figs S8–S10). Stomata in the abaxial sides of the leaves and at the outer sides of pitchers were normal ones with two guard cells (Fig. 3a–c), whereas stomata inside the pitchers were modified ‘lunate cells’, pointing downwards, with only one guard cell (Figs 3d,e and S3). These modified stomata inside the pitcher were found embedded in crystalline epicuticular wax layers (Fig. 3d,e).

### CO_2_, trichomes, prey capture

Leaf abaxial and adaxial sides of *N. khasiana* showed only glandular trichomes (data not shown) at a low density. Branched non-glandular and glandular trichomes were observed on *N. khasiana* tendrils (partially seen in Fig. 3h), at the outer sides of their pitchers and upper sides of their lids (Figs 3b and S11–S13), glandular trichomes only were found in the inner sides of lids (Figs S8–S10), and no trichomes were observed in other inner sides of pitchers (peristome, slippery and digestive zones) (Figs 3d–g and S2–S4).

### Respiration as CO_2_ source within *Nepenthes* pitchers

SEM micrographs of *N. khasiana* tendrils and roots (Fig. 3h–l) showed numerous hollow channels (vascular bundles) within them. Starch granules deposited in root cross sections were also observed in the SEM (Fig. 3l). But, no gas flow was detected from tendril (cross-section) into the pitcher cavities (*Methods, Field studies*). In our comparative measurements, photosynthetic rates (*A*_N_) of *N. khasiana* laminae and pitchers were 3.68 ± 0.53 μmol CO_2_ m^−2^ s^−1^ (n = 6) and −0.60 ± 0.22 μmol CO_2_ m^−2^ s^−1^ (n = 6), respiration rates (*R*_D_) 0.82 ± 0.18 μmol CO_2_ m^−2^ s^−1^ (n = 6) and 1.55 ± 0.36 μmol CO_2_ m^−2^ s^−1^ (n = 6) and maximum quantum yield of PSII (*F*_V_/*F*_m_) 0.80 ± 0.01 (n = 8) and 0.67 ± 0.07 (n = 8), respectively.

### CO_2_, *Nepenthes* pitcher growth, C/N ratio

**Figure 4:**
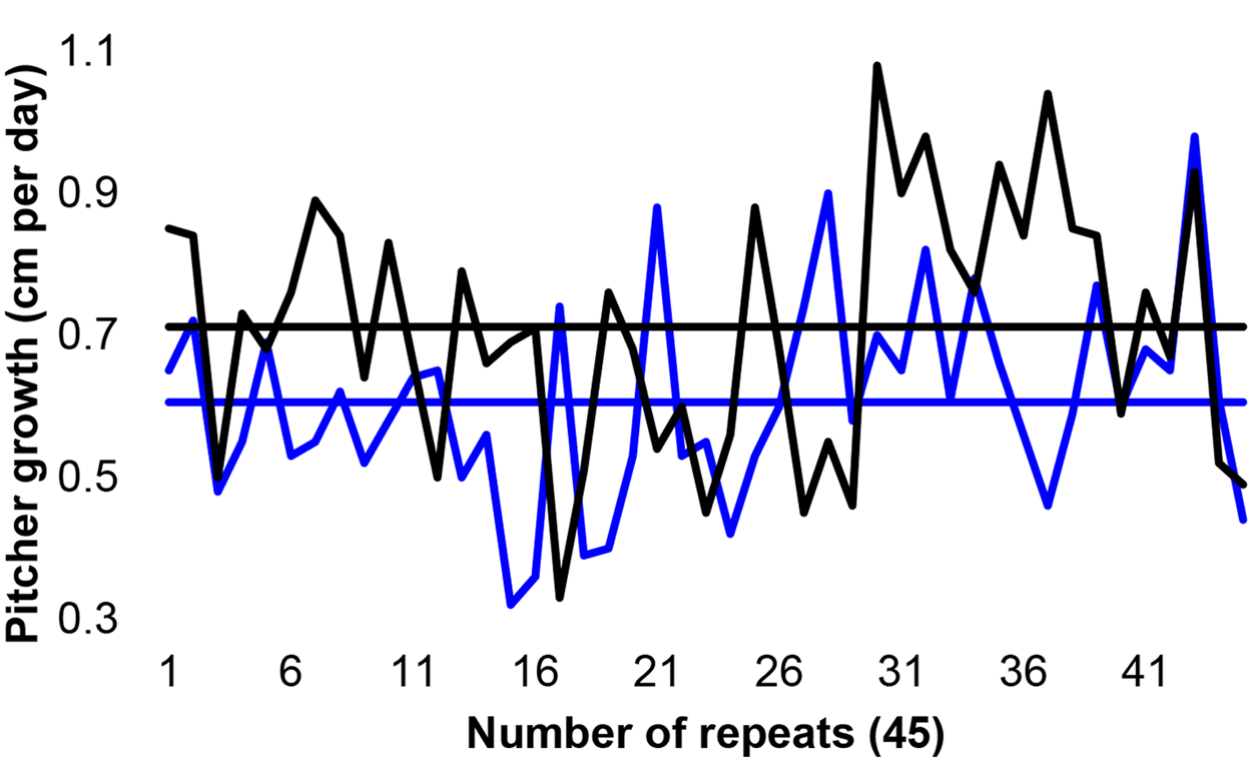
CO_2_ released-*N. khasiana* pitchers show diminished growth rates. Blue, cut pitchers, average growth 0.61 ± 0.14 cm per day, mean ± s.d., n = 45; black, uncut pitchers, average growth 0.71 ± 0.17 cm per day, mean ± s.d., n = 45; straight lines display respective averages (more details in Table S2).

We verified the growth rate of *N. khasiana* pitchers on release of CO_2_ (within them) against normal CO_2_-filled pitchers. *N. khasiana* pitchers in early growth stages, when cut to release the elevated CO_2_ within them showed diminished growth compared to control pitchers. *N. khasiana* cut pitchers: initial stage of 6–8 cm to lid opening, average growth of pitchers 6.84 ± 2.03 cm, n = 45; pitcher growth in cm per day 0.61 ± 0.15, n = 45 (Table S2). *N. khasiana* uncut (control) pitchers: initial stage of 6–8 cm to lid opening, average growth of pitchers 8.00 ± 2.27 cm, n = 45; pitcher growth in cm per day 0.71 ± 0.17, n = 45 (Fig. 4 and Table S2). Growth rate (in cm per day) was diminished by 14.08% in cut pitchers. Growth rate was minimized (to zero) on lid opening of all (cut/uncut) pitchers.

We found high carbon and low nitrogen contents in *N. khasiana* leaves (C 45.72 ± 2.43%, N 2.14 ± 0.30%, n = 4) and pitchers (C 39.07 ± 1.94%, N 1.50 ± 0.25%, n = 4).

## Discussion

Our data demonstrate that *Nepenthes* unopened pitchers are CO_2_-enriched ‘cavities’, when lids open they release CO_2_ at a high 3000–5000 ppm to an ambient ~400 ppm atmosphere, and then continue releasing CO_2_ resulting in its gradient surrounding them. Weight (difference) measurements of *Nepenthes* pitchers indicate the release of a denser gas (CO_2_; density CO_2_/air 1.980/1.225 kg/m^3^) within them, filled at a slightly higher pressure compared to the atmosphere. *Nepenthes* pitchers generally stay in ‘upright’ position, and the gas within is emitted through the pitcher-lid opening.

Open pitchers of *N. khasiana* are constant emitters of CO_2_ (476.75 ± 59.83 ppm, n = 6), a sensory cue. Most insects pay special attention to ‘subtle variations’ or ‘gradients’ of CO_2_ in the form of plumes arising from individual point sources^14, 15^. Insects have well developed CO_2_ receptors which can detect these variations (even small variations) as a means of locating their food^14^. Moreover, CO_2_ emitting devices are widely used as traps against mosquitoes, flies and other insects^16^. In this study, on CO_2_-streaming (1% CO_2_-enriched air) for 12 days through *N. khasiana* pitcher tops, we found a substantial increase in aerial preys (insects) captured within them (preys captured = 31.17 ± 11.91, n = 6), compared to air-streamed control pitchers (same flow rate; preys captured = 16.20 ± 5.15, n = 6) and unmodified (normal) pitchers (preys captured = 19.67 ± 5.43, n = 6) (Fig. 1). These counts are excluding the ants (dead) crawled into these pitchers from the ground, and the ants count did not show any pattern between the CO_2_-enriched, air-streamed and unmodified (normal) pitchers. In Fig. 1, the insect capture rates in these three different experimental conditions (CO_2_-enriched, air-streamed and unmodified pitchers) are proportional to the CO_2_ emission rates from *N. khasiana* pitcher tops. These data demonstrate CO_2_ as an insect attractant emitted by *Nepenthes* prey traps, and reveals a new prey capture mechanism within them.

Most *Nepenthes* species secrete pitcher fluids with viscoelastic properties. Fluids in unopened pitchers are sterile^17^, and once open microbes and inquilines invade them. Our results show that a high level of CO_2_ is dissolved in *N. khasiana* pitcher fluids (Fig. 2). Open, prey captured pitcher fluids showed low levels of O_2_ (Fig. 2), and very low (even anoxia) or decreasing levels of oxygen were reported in *Sarracenia purpurea*, *Utricularia* and *Genlisea* traps^18–20^. Dissolved CO_2_ in *Nepenthes* pitcher fluid instantaneously forms equilibrium with its hydrated form H_2_CO_3_ which dissociates into H^+^ and HCO_3_^− 21^. The relative changes to any one of these molecules/ions control the pH and optimum activity of the digestive enzymes secreted into the pitcher fluid by specialized glands^21^ (Fig. 3f,g). *N. khasiana* pitcher fluids are acidic, and lid opening and prey capture further reduce the pH. Similar pH trends were observed in pitcher fluids of several *Nepenthes* species^4, 5, 11, 22^. pH reduction on prey capture (even after release of the high levels of CO_2_ on pitcher opening) is critical for optimum enzyme activity (prey digestion) and absorption of nutrients, and this is achieved through a proton (H^+^) pump^22–24^. This acidic pH could also be controlling the growth of pitcher inhabitants (microbes, mosquito larvae, small aquatic organisms etc.). CO_2_ dissolved in the pitcher fluid is one of the factors making it acidic and it also acts as a preservative to the pitcher fluid.

Once *Nepenthes* pitchers become mature, their ‘tight lid sealings’^1^ open and release the elevated CO_2_ within, making them ready for prey capture. The sequential events of lid opening, CO_2_ release and prey capture are sensed by these plants, and they release antifungal naphthoquinones (droserone, 5-O-methyl droserone, plumbagin, 7-methyl juglone) into the pitcher fluid (Figs S5–S7), preventing infections from incoming preys^5^.

Stomata are small pores controlling gas exchange, mainly CO_2_ and water vapour, found in leaves and other organs in plants^25^. Stomata inside *N. khasiana* pitchers were ‘modified’, pointing downwards, with only one guard cell (Figs 3d,e and S3). Similar modified stomata embedded in wax crystals were observed by SEM studies in the inner sides of pitchers of *N. rafflesiana* ^11, 12, 22^, *N. alata* ^1, 3, 26^, *N. mirabilis* ^3^, *N. diatas* ^23^ and other *Nepenthes* species/hybrids^27, 28^. Most authors described these stomata as ‘transformed’ or as ‘lunate cells’ with a convex structure in the inner surface of *Nepenthes* pitchers, and explained this modification as an evolutionary adaptation contributing to prey capture by disrupting the adhesion of insect feet and blocking entry of their claws^12, 22, 23, 26^. Owen and Lennon, 1999 suggested the function of this ‘modified stomatal complex’ as ‘water secretion’ or ‘gas exchange’ or even as a ‘mystery’^1^. But, absence of ‘pores’^1, 22^ in these ‘modified stomatal structures’ nullifies the chances of them functioning as vents in ‘gas exchange’.

Similar to our observations, Pavlovič and co-workers reported stomata on the abaxial sides of laminae of *N. alata* and *N. mirabilis*, and very low stomatal density in *Nepenthes* pitchers^3^. Other studies also reported modified stomata at the interior of pitchers and overall low stomatal density in pitchers of various *Nepenthes* species^1^. Stomatal distribution in laminae (abaxial, high) and pitchers (stomata with two guard cells, outer side; low) are matching with their photosynthetic capacities, high (laminae) and very low (pitchers). In most cases, we found high density of the ‘modified stomata’ at the pitcher inner (top) sides (Fig. S3)^1, 9, 23^. In *Nepenthes*, pitchers are formed by the folding of leaves with their adaxial (upper) surfaces turning into inner sides of these traps^1^. It is significant that, the leaf upper surfaces are devoid of stomata, but the pitcher inner surfaces ‘evolved’ these ‘modified stomata’ (Fig. 3d,e). In pitchers of *Sarracenia*, *Darlintonia*, *Heliamphora* and *Cephalotus*, stomata (normal) are found in their outer surfaces or in their lids/‘hoods’, and ‘stomata-like structures’ present within their pitcher tubes are ‘permanently open’ and not ‘functional’^12^. It is proven that increase in CO_2_ even in the range of 100 ppm has a profound effect on the stomata (modifies their morphology) in plants^14^. The transformed stomatal aperture with a single guard cell (Fig. 3d,e) at the interior (only) of *Nepenthes* pitchers is most probably a manifestation of the high CO_2_ (approx. 4000 ppm, nearly 10 times the ambient) atmosphere within them. But, evidences gathered so far are not conclusive on the function of these ‘modified stomata’ or ‘lunate cells’ (Figs 3d,e and S3)^1, 12^. Crystalline epicuticular wax in thick layers, as observed in the upper part of inner pitcher walls of *N. khasiana* and several other *Nepenthes* species, is not distinctly seen in other portions of the pitchers (lid, peristome, liquid zone, outer surface) and in the abaxial and adaxial sides of their leaves (Fig. 3). These inner waxy layers define the hydrophobic slippery zone, which minimizes insect attachment. Recent evidences also demonstrate high level of CO_2_ as a factor which enhances cuticular wax density in plants^29^. *Nepenthes* prey traps display a unique natural model of evolution of stomata in a CO_2_-enriched atmosphere.

Trichomes, a group of epidermal microstructures, carry out diverse functions in plants, and in carnivorous plants one of their roles is facilitating prey capture^30, 31^. In fact, relatively high density of branched trichomes was observed at the top outer sides *N. khasiana* pitchers and their lids^1^ (Figs S11–S12), and no trichomes were observed in deep interior of the pitchers. But, significantly, *Sarracenia*, *Heliamphora*, *Darlingtonia* and *Cephalotus* pitchers have trichomes in their interior zones, including their innermost digestive zones^1, 12, 32^. Branched trichomes on the exterior of *Nepenthes* pitchers (and their lids) provide a foothold to the visitors (termites, ants etc.)^30, 31^, enhancing the chances of their ultimate ‘luring’ to the interior of the traps. Edible trichomes in *N. albomarginata* are known to ‘lure’ termites into their pitcher traps^23, 33, 34^. Elevated CO_2_ within *Nepenthes* traps could be one factor reducing the trichome density (particularly branched ones) in the inner sides of *Nepenthes* pitchers^35^.

SEM micrographs showed numerous vascular bundles within the roots and tendrils of *N. khasiana* (Fig. 3h–l), but no gas flow was detected from tendril (cross-section) into the pitcher cavities. Respiration (dark) rates of non-carnivorous herbaceous plants are typically less than 50% of their photosynthetic rates, but, the average respiration/photosynthetic rate in terrestrial carnivorous plants is as high as 63%^36^. Again, the traps (pitchers, snap trap) of terrestrial carnivorous plants (*Nepenthes*, *Sarracenia*, *Dionaea muscipula*) showed much higher respiratory costs (respiration/photosynthetic rate 158%) than their laminae (lamina, phyllodia, petiole) (respiration/photosynthetic rate 19%)^36^. More evidences for higher respiration rates (in traps compared to laminae) are available in carnivorous plants with ‘active’ trapping mechanisms (*D. muscipula*; *Utricularia*, bladder traps)^36–38^. Our results show that, *N. khasiana* laminae have significantly higher photosynthetic capacity compared to their pitchers whereas respiration rates are comparatively high in pitchers. Similarly, maximum quantum yield of PSII (Fv/Fm) in *N. khasiana* laminae is high compared to their pitchers. These parameters are matching with similar previous measurements in other *Nepenthes* species^39^. Unlike most plant leaf structures, high growth rate and unique physiological functions (prey attraction, capture, digestion, absorption of nutrients) of *Nepenthes* pitchers demand more energy, prompting higher respiration rates in the trap tissues, resulting in the release of more of CO_2_. Carnivorous plants follow the C3 photosynthetic pathway, and high CO_2_ levels are also known to enhance respiration rates in C3 plants^40^. Thus, we demonstrate respiration of pitcher tissues as the factor contributing to the high CO_2_ within the ‘closed cavities’ of *Nepenthes* traps.

*Nepenthes* tendrils and pitchers grow at a faster rate from their leaf terminals. ‘Rapid elongation’ of growing *Nepenthes* pitchers and their limited growth after opening of the lid sealing were previously observed by other authors^1^. Owen and Lennon, 1999 found a uniform growth rate of 0.0147 ± 0.0001 cm per h (0.35 cm per day) for *N. alata* pitchers, from initiation to the point of lid opening^1^. A small incision on defined *N. khasiana* pitchers (initial length, 6–8 cm) released the high CO_2_ within them, and these pitchers continued growth at a diminished rate compared to control pitchers (Fig. 4). In control (uncut) pitchers, the balancing of CO_2_ levels (with atmosphere) occurs only on lid opening. Our data indicate that, as in other CO_2_-enrichment studies, elevated (entrapped) CO_2_ within acts as a growth promoter of *Nepenthes* prey traps. Recent studies revealed key data/facts on comparative anatomy^41^ and construction costs^42^ of leaves/pitchers of *Nepenthes* species, leaf development in *S. purpurea* ^43^ and the influence of CO_2_ on leaf phenology in plants^44^. More investigations, in the light of the discovery of CO_2_ within, could possibly unravel similar growth patterns (tissue specific changes in cell division)^43^ and faster growth rates in *Nepenthes* pitchers. Carbon contents of *N. khasiana* leaves are comparable to those of non-carnivorous plants^3, 42^, but, both C and N contents are comparatively low in the pitchers^42^. As in other *Nepenthes* species^3, 45, 46^, the C/N ratio of *N. khasiana* pitchers is high, 26.05 (n = 4).

CO_2_ (high) and CO, CH_4_ and N_2_O (ambient) found in *Nepenthes* pitchers are greenhouse gases. Global CO_2_ levels are predicted to go up to 800 ppm by 2100 and further onto even higher levels^14^. *Nepenthes* prey traps with elevated CO_2_ contents (3000–5000 ppm) are simulating this futuristic scenario in their ‘closed cavities’ (before trap opening). As in other CO_2_-enrichment experiments^14^, high carbohydrate and low protein contents were detected in *Nepenthes* pitchers^3^. Carbohydrate accumulation is a major acclimation response to elevated CO_2_^14^. High carbohydrate contents in pitchers, transformed into nectar by nectaries (Figs S3 and S4), act as a major ‘lure’ in prey capture. Chlorophyll content is generally low in pitchers compared to their laminae. In some *Nepenthes* species, pitchers are red-tinted indicating low chlorophyll contents (Fig. S1). Pitchers in *Nepenthes* have very low photosynthetic rates compared to their laminae^3^. Reduction in photosynthetic rates in *Nepenthes* pitchers is primarily due to factors such as replacement of chlorophyll-containing cells with digestive glands, low nitrogen, chlorophyll contents and low stomatal density^3, 14^. Photosynthetic Nitrogen Use Efficiency (PNUE) is also significantly low in *Nepenthes* pitchers compared to their laminae. Recently Pavlovič and Saganová pointed out reduced Rubisco activity in *Nepenthes* prey traps^39^, and Rubisco content is known to decrease with elevated CO_2_. These factors viz., photosynthetic rate, C/N ratio, carbohydrate/protein contents, chlorophyll content and PNUE, of several *Nepenthes* species were compared between their laminae and pitchers by various groups (*N. alata* and *N. mirabilis* ^3^, *N*. *talangensis* ^47^, 8 *Nepenthes* species and hybrids^42^ and 15 carnivorous plants including *Nepenthes* hybrids^46^). These parameters of *Nepenthes* leaves and pitchers were also compared to non-carnivorous plants^45, 46^.

These trends in *Nepenthes* pitchers mainly, burst of growth, enhanced carbohydrate levels, declined protein levels, drop in photosynthetic capacity, high respiration rate and evolved stomata, are probable manifestations of the enhanced CO_2_ atmosphere within them. These evidences also infer *Nepenthes* pitchers as ideal examples reflecting the effects of an anticipated high CO_2_ level on Earth’s surface, on the characteristic features of plants. Recently, several groups put forward ‘construction cost or cost/benefit theories’^3, 42, 45, 46^ on *Nepenthes* prey traps. Most of these studies estimated the nutritional benefit gained from captured preys above (at least marginally) the cost of constructing traps by leaf modification. Future construction cost estimates need to take into account of the acclimation responses of *Nepenthes* pitchers due to the ‘so far unknown factor’ of high CO_2_ content within them.

In conclusion, *Nepenthes* pitchers are CO_2_-enriched cavities, and CO_2_ emission from open pitchers acts as a sensory cue attracting insects towards these traps. Most of the characteristic features of *Nepenthes* pitchers are influenced by the high content of CO_2_ entrapped within them. This study also hypothesizes *Nepenthes* pitchers as natural model systems mimicking an anticipated elevated CO_2_ scenario on Earth.

## Methods

### *Nepenthes* pitchers, gas sampling

*N. khasiana* mature, unopened pitchers (Fig. S1a) were collected from three established populations (08°45′ 00.05″N77°01′45.35″E, altitude 110 m; 08°45′00.04″N, 77°01′41.09″E, altitude 112 m; 08°44′59.74″N, 77°01′40.31″E, altitude 112 m) in Jawaharlal Nehru Tropical Botanic Garden and Research Institute (JNTBGRI) garden sites and the gas compositions inside them were analyzed by gas chromatography (GC-FID/ECD/TCD). Gas compositions inside mature, unopened pitchers of various *Nepenthes* hybrids (Fig. S1b–g) grown in a greenhouse (08°45′14.59″N, 77°01′31.37″E, altitude 106 m) at JNTBGRI campus were also tested. Gas samples from inside the opened *N. khasiana* pitchers (from inside, below the peristome) were collected using syringes (Dispovan, Hindustan Syringes and Medical Devices Ltd., Faridabad, India) with a three way stop cock (IGNA, Ignisol Mediplas-Corp, Mumbai, India) and subjected to gas chromatographic analysis. Air samples from JNTBGRI campus were also analyzed.

Lids of mature (about to open, red colour appears at the peristome portion) *N. khasiana* pitchers in the field were sealed with super glue (to prevent lid opening). Then a small ‘cut’ (average 5.4 × 5.7 mm) was made on the top half (above liquid zone) of the pitcher (for gas release). After 24 h, the cut portion was sealed with parafilm/super glue. After 2 days of sealing, pitchers were collected and subjected to gas analysis. In another set of experiments, lids of opened *N. khasiana* pitchers (opened a day before) were sealed back with super glue. After 2 days of sealing, these pitchers were collected and their gas compositions were analyzed.

### Gas analysis by GC-FID/ECD/TCD

*N. khasiana*/*Nepenthes* hybrid unopened pitchers were opened underwater and the gases inside pitchers were collected by the displacement of water. This is to avoid possible mixing with air and dilution of the contents of the pitchers, when opened in air. The gases from the pitchers were transferred to syringes and analyzed through gas chromatography. A Clarus 580 gas chromatograph (Perkin Elmer, Waltham, USA) equipped with a Flame Ionization Detector (FID) and an Electron Capture Detector (ECD) was used. FID had a Methanator for converting CO and CO_2_ to methane. ECD measured nitrous oxide in the sample. A gas sampling valve with 100 µl sampling loop was used for injecting the sample to the column. Isothermal separation was achieved at 35 °C in an Elite-PLOT Q column (30 m × 0.53 mm) with nitrogen carrier gas. Another NUCON 5765 gas chromatograph (Aimil, New Delhi, India) with a Thermal Conductivity Detector (TCD) and packed column (PORAPAK Q, 80/100 mesh, 5 m long) with nitrogen as carrier gas was used for the measurement of oxygen in the samples. FID, Methanator and ECD were calibrated with the standard gas mixture containing CH_4_, CO_2_, CO and N_2_O in nitrogen gas.

### Head space GC/MS/MS of *N. khasiana* pitcher fluids

*N. khasiana* pitcher fluids (3 mL each) and 20 mL standard CO_2_ (carbon dioxide-N5.0, certified concentration 5.49%, nitrogen-N-5.0 balance, Chemtron Science Laboratories, Mumbai, India) bubbled into 3 mL distilled water were transferred to the head space unit (separately) and analyzed by GC/MS/MS. Injection mode: GC head space (Combi Pal, CTC Analytics, Switzerland), syringe temperature 50 °C, sample agitator temperature 60 °C, incubation time 5 min. GC: CP-3800 (Varian, CA, USA), VF-5 (5% phenyl 95% dimethyl polysiloxane, non-polar, 30 m × 0.25 mm i.d., 0.25 μm film thickness) capillary column, column temperature programme isothermal 60 °C for 20 min, flow rate 0.5 mL min^−1^, MS: Saturn 2200 GC/MS/MS (Varian, CA, USA), mass range 20–60 m/z.

### Partial pressures of CO_2_, O_2_ in *N. khasiana* pitcher fluids

Partial pressures of CO_2_ and O_2_ in *N. khasiana* (mature, unopened and opened, prey captured) pitcher fluids were determined using a calibrated ABL800 Basic Gas Analyzer (Radiometer, Copenhagen, Denmark) (Fig. 2).

### SEM of *N. khasiana* roots, leaves, tendrils and pitchers

SEM analyses of *N. khasiana* abaxial/adaxial sides of leaves, inner/outer sides of pitchers, lids, tendril and roots were carried out on a S-2400 Scanning Electron Microscope (Hitachi, Tokyo, Japan) (Figs 3, S2–S4, S8–S13). *N. khasiana* samples were fixed with 3% gluteraldehyde in phosphate buffer and kept overnight. Samples were then dehydrated sequentially with 30%, 50%, 70% ethanol (15 min each, two changes) and 90%, 100% ethanol (30 min each, two changes). These dehydrated samples were subjected to critical point drying, coated with gold and viewed on the SEM.

### DART-MS of *N. khasiana* pitcher fluids

Pitcher fluids (yellow coloured) from prey captured *N. khasiana* pitchers (Fig. S5), chitin induced^5^ (Fig. S6) and uninduced (colourless on opening, before prey capture) pitchers (Fig. S7) were collected, lyophilized and analyzed on an AccuTOF JMS-T100LC Mass Spectrometer having a DART (JEOL, MA, USA). Samples were analyzed directly in front of the DART source. Dry He was used at a flow rate of 4 L min^−1^ for ionization at 350 °C. Orifice 1 was set at 28 V, spectra were collected, and the data from 6–8 scans were averaged.

### *Nepenthes* pitcher weight measurements

*N. khasiana* and *Nepenthes* hybrid (mature, unopened) pitchers were collected and their fresh weights were recorded. Then, pitchers were cut open just above the pitcher fluid level (to release the entrapped gas) and the entire pitcher contents were (very) quickly re-weighed (Table S1).

### Field studies

*N. khasiana* pitchers were covered (netted) with colourless nets to prevent ants and insects entering on lid opening. Netting was done a week before opening on near mature pitchers. Three days after opening pitcher fluids were collected, lyophilized and analyzed.

CO_2_-enriched air (1% CO_2_ in air; Bhuruka Gases Ltd., Bangalore, India) was passed into just opened *N. khasiana* pitchers in the field through a small cut made above the fluid level by inserting a long, colourless tubing (inner diameter 2 mm; average flow 25.72 mL/min), and prey (aerial) capture was monitored for 12 days. Similarly, air at the same flow rate was streamed through control pitchers. On the 6th day, gas samples from inside test/control pitchers (just below the peristomes) were collected in syringes and analyzed by gas chromatography (n = 6, each). On the 12th day after lid opening, the entire contents of test/control *N. khasiana* pitchers (n = 6, each) were (separately) transferred to petri dishes (Fig. 1), and captured aerial preys (in each dish) were carefully counted. Similarly, prey (aerial) capture rates in normal (unmodified) pitchers (with no CO_2_/air streaming) in 12 days were also counted. In all three experiments, ants (dead) crawled into these pitchers from the ground were not considered (counted).

Tendrils of live *N. khasiana* plants were cut just below the pitchers and their cross sections were inserted into inverted syringes partially filled with water (for 6 days) in the field. On repeated experiments, no gas bubbling or any other changes in the water were observed.

### *N. khasiana* pitcher growth measurements

*N. khasiana* pitchers from the three populations in JNTBGRI garden sites with an initial growth of 6 to 8 cm were marked, their initial pitcher lengths were noted and small cuts (average 5.4 × 5.7 mm, to release the gas inside pitchers) were made above the fluid level. These test pitchers were constantly monitored, pitcher lengths on the day of lid opening and the number of days required till lid opening (from an initial stage of 6 to 8 cm) were noted. Similar measurements were also made on control *N. khasiana* pitchers (with no cuts) (Fig. 4 and Table S2).

### C, N contents in *N. khasiana* leaves, pitchers

*N. khasiana* leaves and pitchers were dried at 60 °C for 72 h and their carbon and nitrogen contents were analyzed on a Vario EL III CHN Analyzer (Elementar Analysensysteme GmbH, Hanau, Germany).

### Chlorophyll-A fluorescence, photosynthesis (A_n_) and dark respiration (R_d_) of *N. khasiana* laminae and pitchers

Chlorophyll-a fluorescence kinetics, *A*_N_ and *R*_D_ of *N. khasiana* laminae and pitchers were measured using a LI-COR 6400 XT portable infrared analyzer (LI-COR, Lincoln, NE, USA), equipped with a leaf chamber fluorometer. Laminae and pitchers from four *N. khasiana* plants in the field were subjected to these measurements. Fully grown *N. khasiana* laminae and healthy, prey captured pitchers (pitcher walls and lids were directly placed into the cuvette, independently) were taken for measurements. A constant PAR (photosynthetically active radiation) of 800 μmol m^−2^ s^−1^ of red (90%) and blue (10%) light was chosen as actinic light intensity and the measurement of chlorophyll fluorescence and *P*_N_ were at ambient CO_2_ level, temperature (33 ± 1 °C), RH (relative air humidity) ~80% and air flow rate of 300 μmol s^−1^. *R*_D_ was measured under similar conditions, except that the plant samples were under dark conditions. The laminae and traps were kept in the chamber for 5–10 min, until steady state of CO_2_ concentrations were reached. Vapor pressure deficit in the sample cell ranged between 0.7 and 1.3 kPa. Minimal fluorescence (*F*_0_) was measured for overnight dark adapted plant samples whereas maximal fluorescence (*F*_m_) was recorded at a PAR of 8000 μmol m^−2^ s^−1^ (saturating flash). Maximal quantum yield of PSII was calculated as *F*_v_/*F*_m_ = (*F*_m_ − *F*_0_)/*F*_m_.

### Statistical analysis

Prey capture rates (Fig. 1), partial pressure measurements (Fig. 2), pitcher size/weight measurements (Table S1) and growth parameters of cut/uncut pitchers (Fig. 4 and Table S2) are expressed as mean ± s.d. Statistical comparisons were done using student’s t-test (Figs 1 and 2). Values of *p* < 0.05 were considered as statistically significant.

### Data availability

All data generated or analyzed during this study are included in this published article (and its Supplementary Information file).

## Electronic supplementary material

Supplementary Information
