# Supplementary material for: Nepenthes pitchers are CO2-enriched cavities, emit CO2 to attract preys
Source: Sci Rep. 2017 Sep 12;7:11281. doi: 10.1038/s41598-017-11414-7 (PMC5595901; doi:10.1038/s41598-017-11414-7)
Supplement: Supplementary file 1 — Supplementary Information [file 41598_2017_11414_MOESM1_ESM.pdf]

Supplementary information

Nepenthes pitchers are CO_2-enriched cavities, emit CO_2 to attract preys

Sabulal Baby[1,*], Anil John Johnson[1], Elavinamannil Jacob Zachariah[2] & Abdul Azeez Hussain[3]

[1]Phytochemistry and Phytopharmacology Division, Jawaharlal Nehru Tropical Botanic Garden and Research Institute, Pacha-Palode, Thiruvananthapuram 695 562, Kerala, India; [2]Atmospheric Sciences Division, National Centre for Earth Science Studies, Post Box No. 7250, Akkulam, Thiruvananthapuram 695 011, Kerala, India; [3]Garden Management Division, Jawaharlal Nehru Tropical Botanic Garden and Research Institute, Pacha-Palode, Thiruvananthapuram 695 562, Kerala, India

Correspondence and requests for materials should be addressed to S.B. (sabulal@jntbgri.res.in; sabulal@gmail.com)

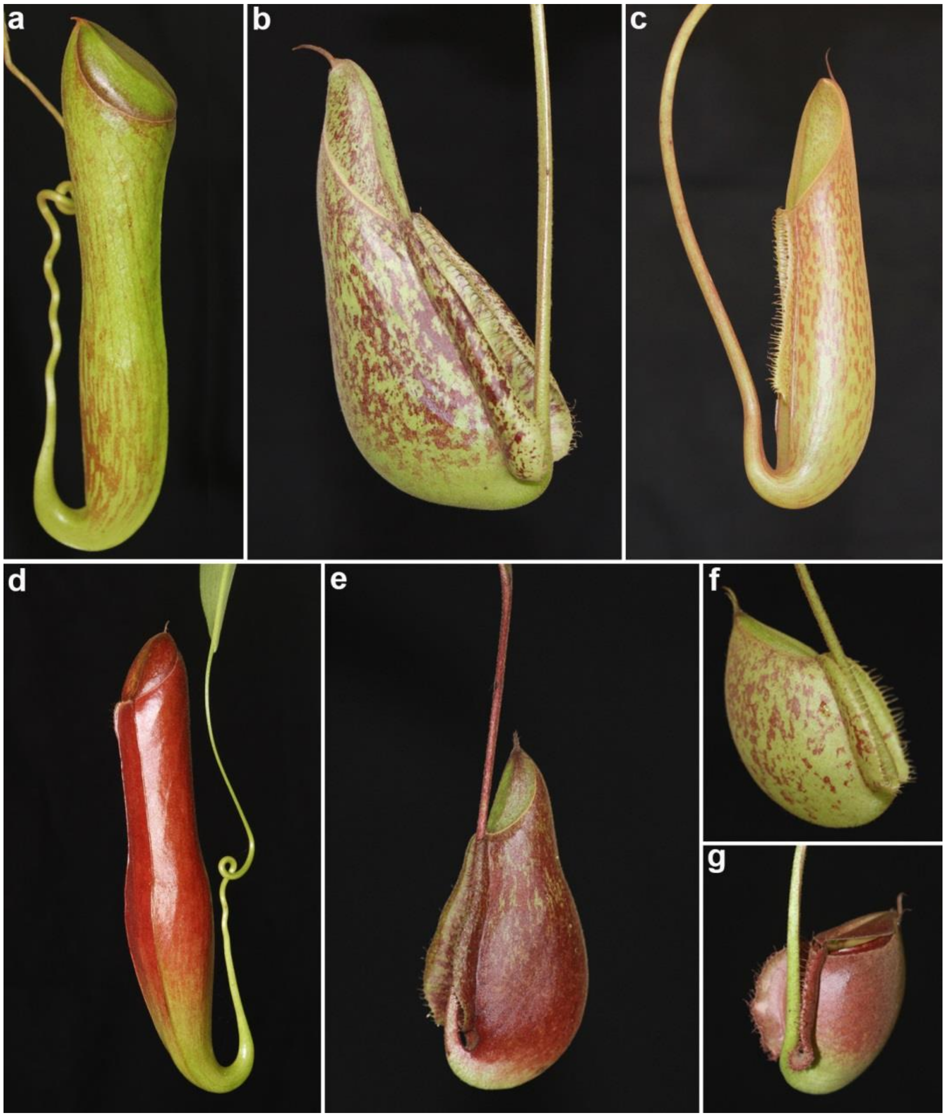

Fig. S1**|** Pitchers of *Nepenthes khasiana* and *Nepenthes* hybrids, with their tendrils. **a**, *Nepenthes khasiana*. **b**, *Nepenthes* hybrid 01 (NH01). **c**, *Nepenthes* hybrid 02 (NH02). **d**, *Nepenthes* hybrid 03 (NH03). **e**, *Nepenthes* hybrid 04 (NH04). **f**, *Nepenthes* hybrid 05 (NH05). **g**, *Nepenthes* hybrid 06 (NH06).

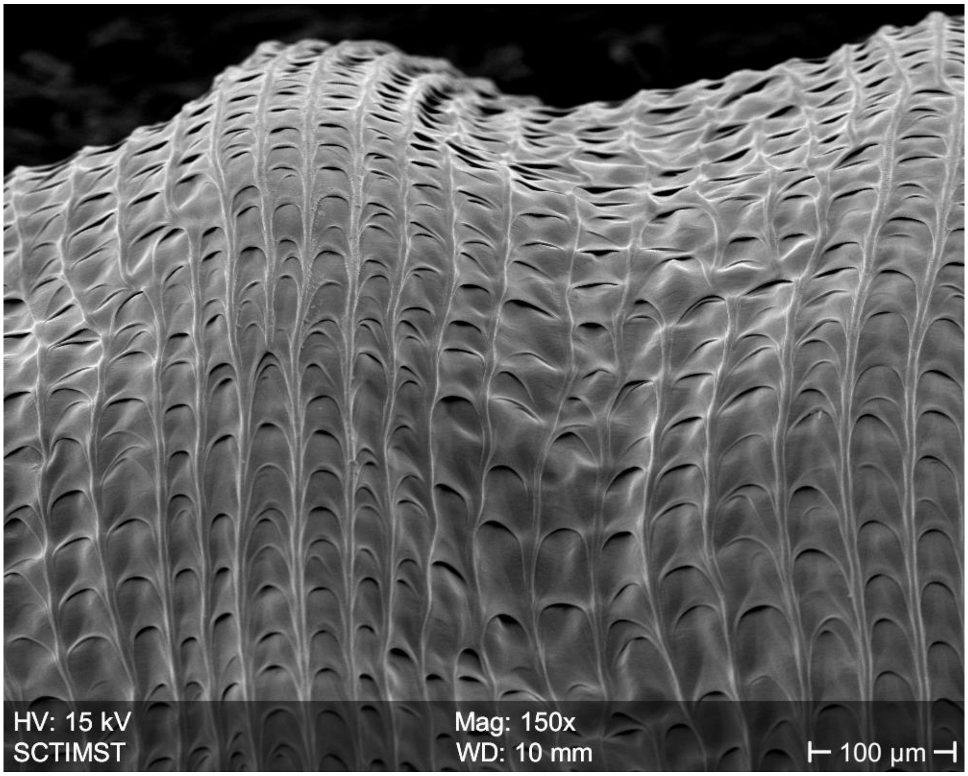

Fig. S2**|** SEM image of *N. khasiana* peristome, showing anisotropic arrangement of the microgrooves.

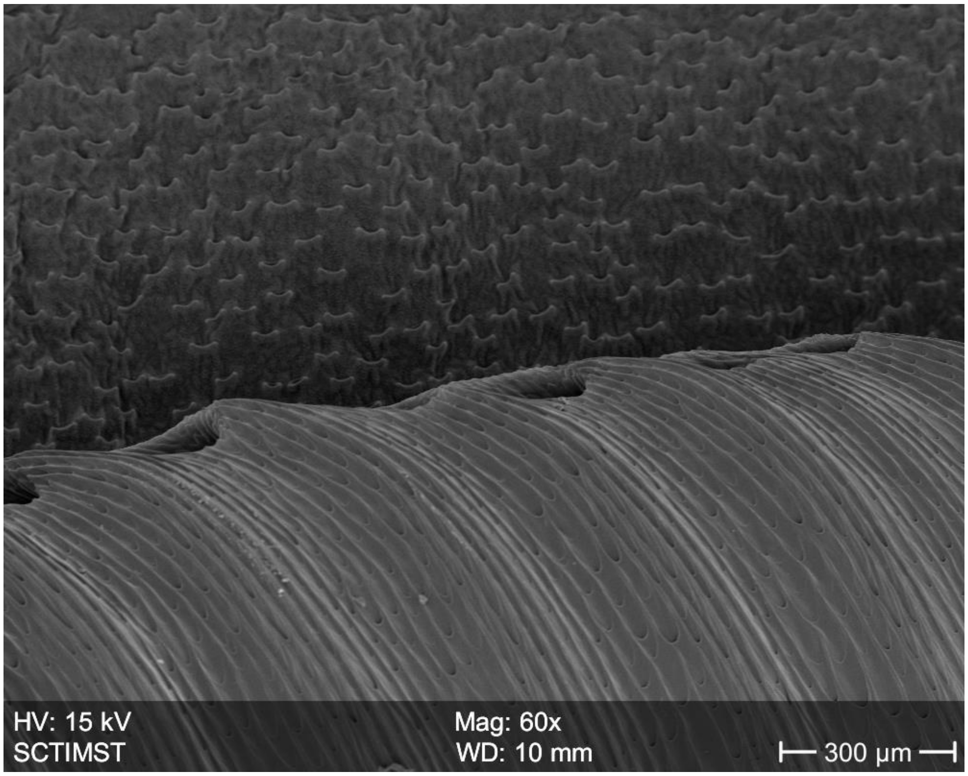

Fig. S3| SEM image of *N. khasiana* peristome inner edge, showing anisotropic arrangement of the microgrooves, nectaries and the high density of modified stomata.

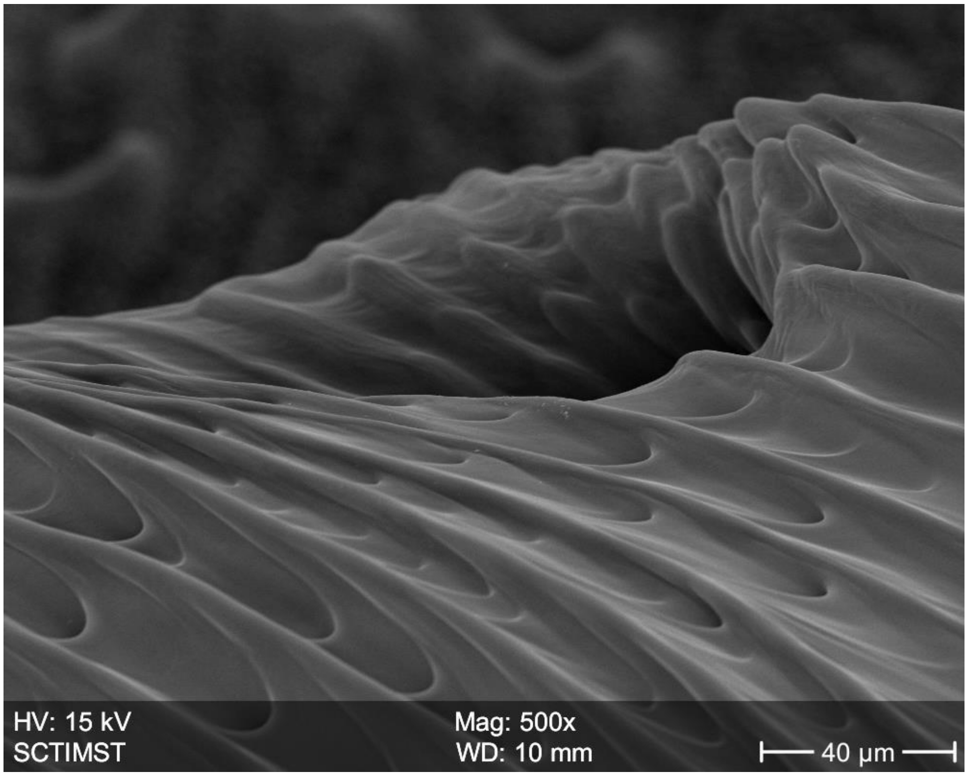

Fig. S4**|** SEM image of *N. khasiana* peristome inner edge, showing a nectary immersed in the microgrooves.

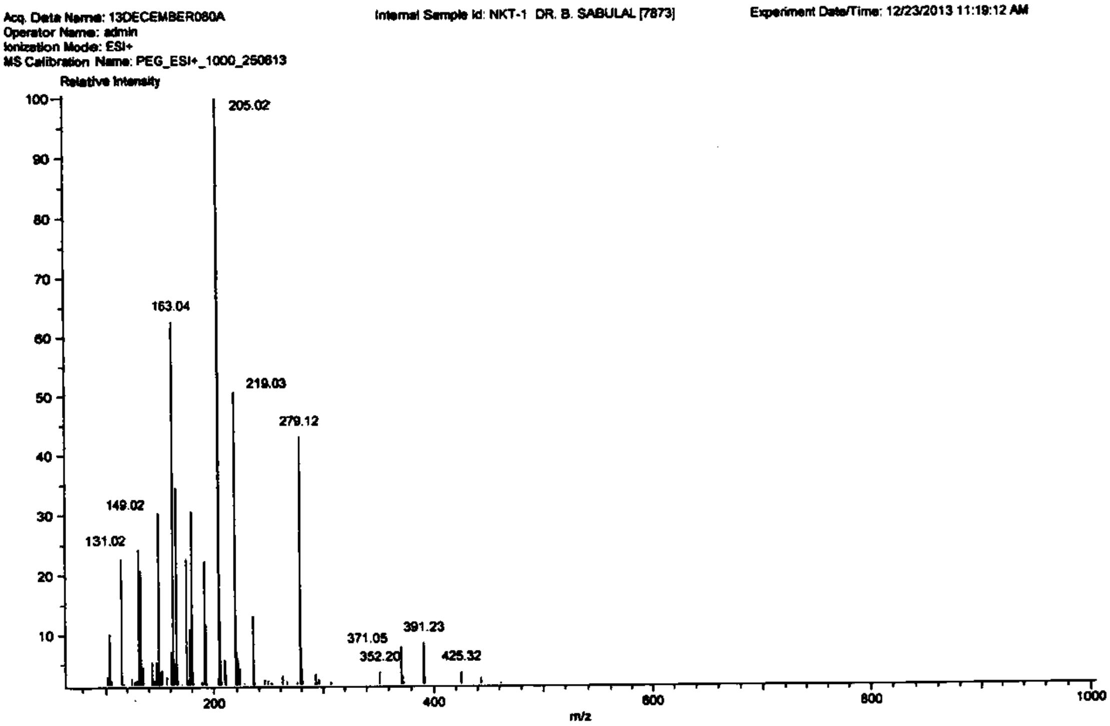

Fig. S5| DART-MS of prey captured (yellow coloured) pitcher fluid of *N. khasiana*.

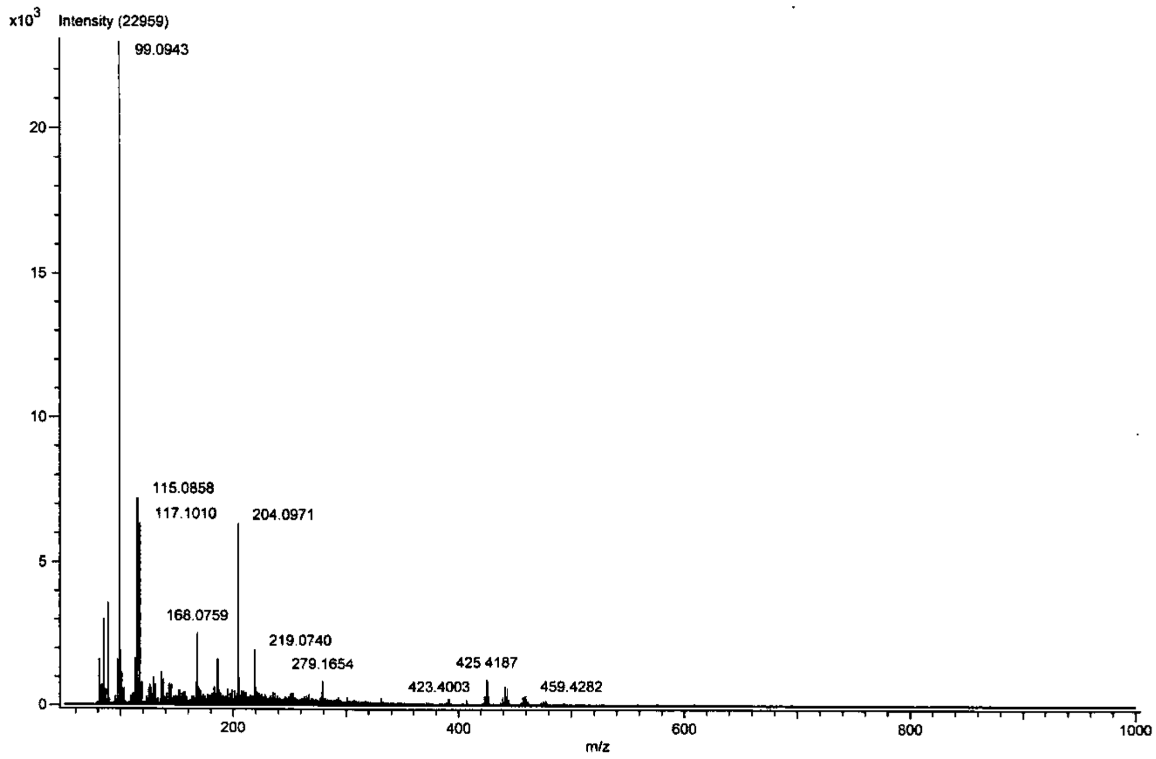

Fig. S6| DART-MS of chitin induced (yellow coloured) pitcher fluid of *N. khasiana*.

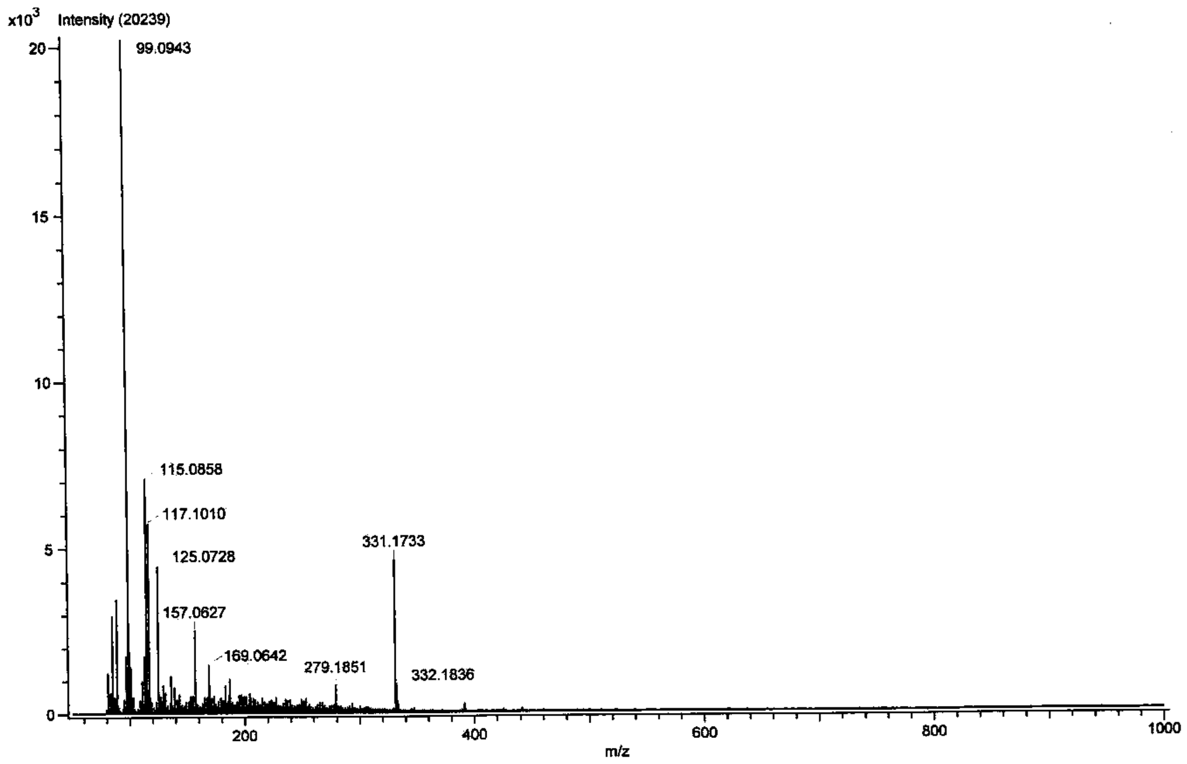

Fig. S7**|** DART-MS of normal (colourless, before prey capture) pitcher fluid of *N. khasiana*.

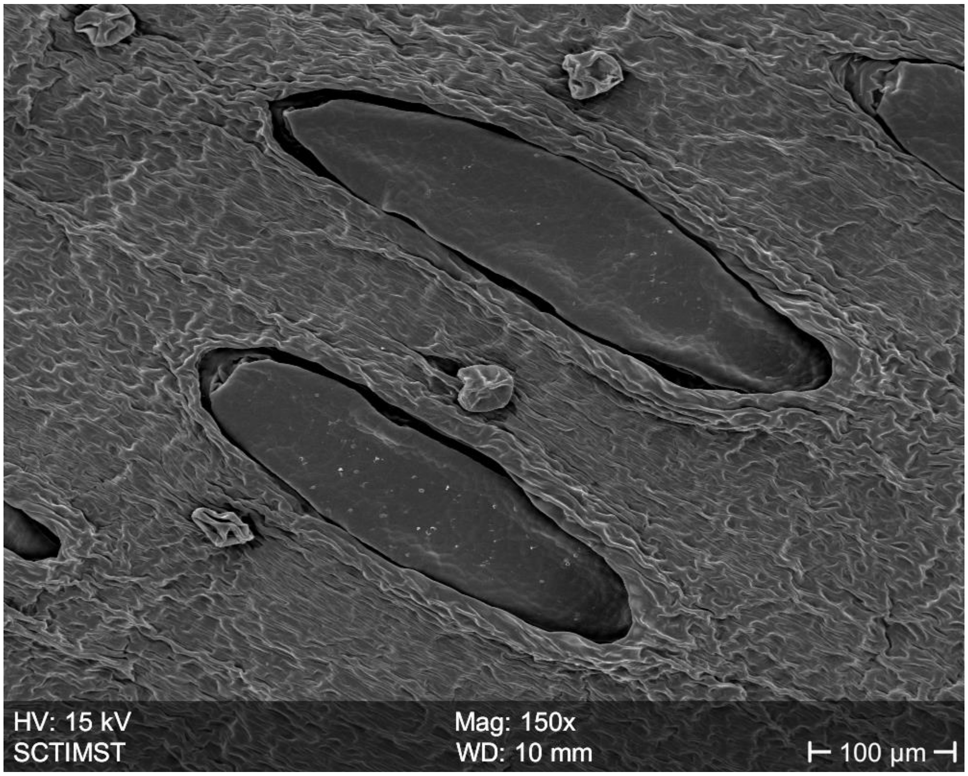

Fig. S8| SEM image of the underside of *N. khasiana* pitcher lid, showing nectar glands and trichomes[1,2].

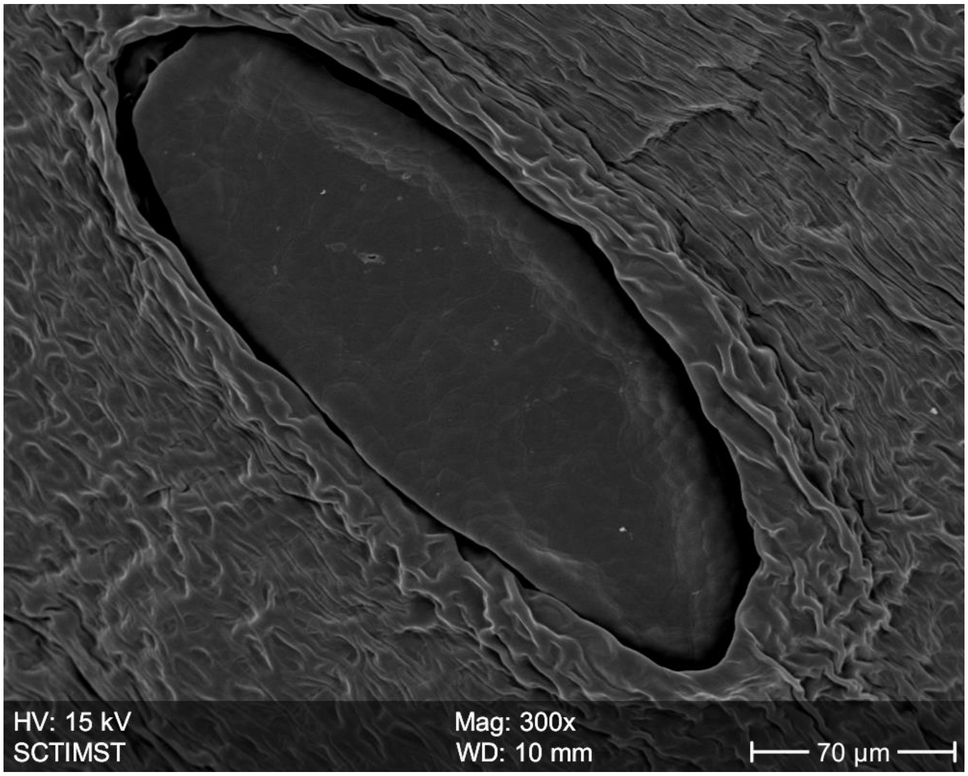

Fig. S9**|** SEM image of *N. khasiana* pitcher lid underside, nectar gland (expansion)[1,2].

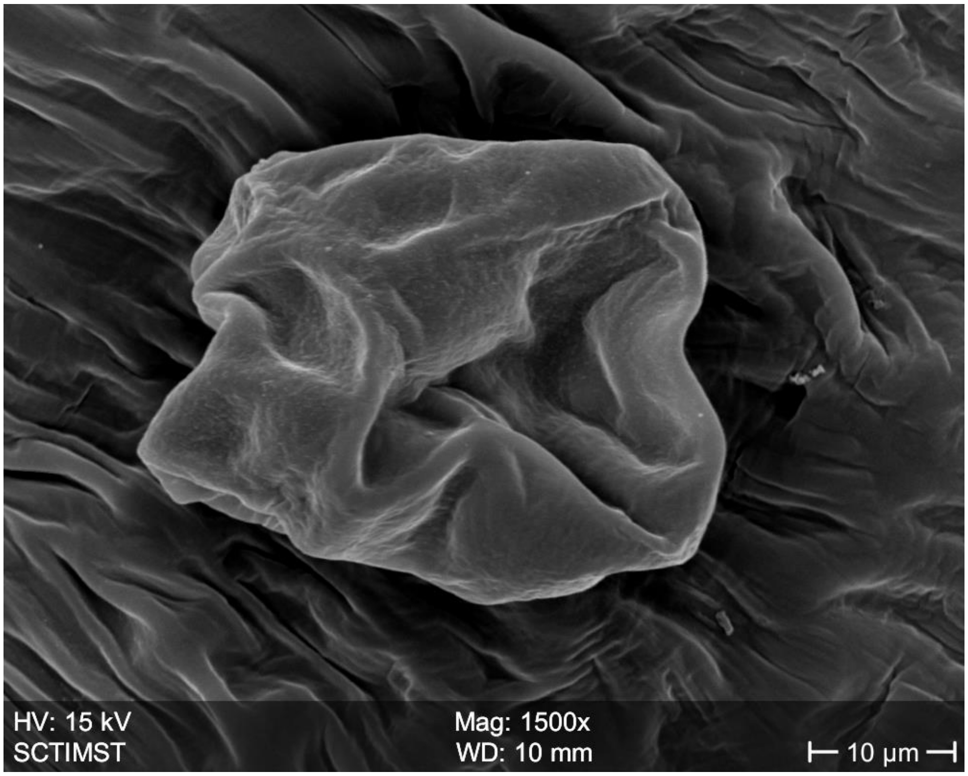

Fig. S10| SEM image of *N. khasiana* pitcher lid under side, glandular trichome (expansion)[1,2].

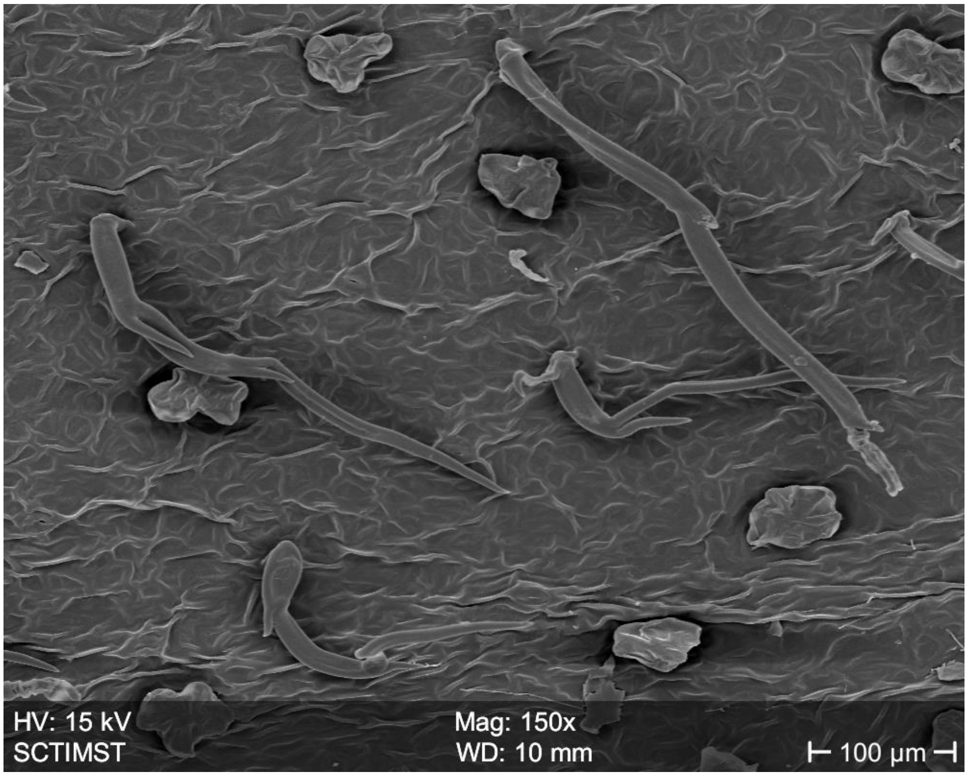

Fig. S11**|** SEM image of *N. khasiana* pitcher outer side (middle), branched non-glandular and glandular trichomes[1,2].

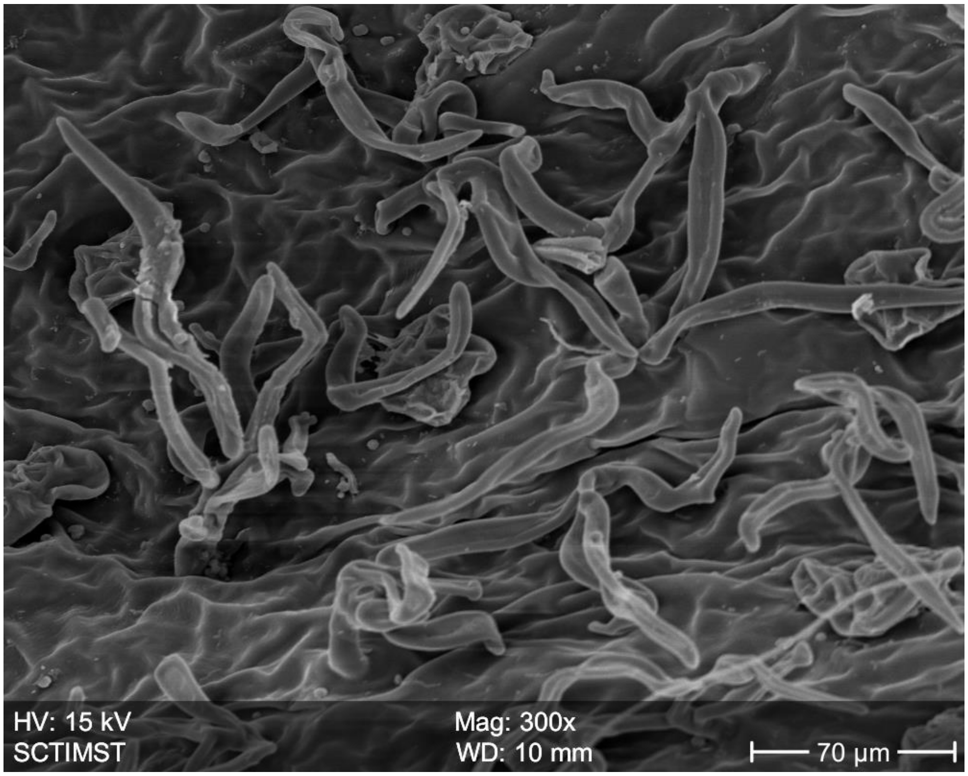

Fig. S12**|** SEM image of *N. khasiana* pitcher outer side (top, just below the peristome), branched non-glandular and glandular trichomes at higher density[1,2].

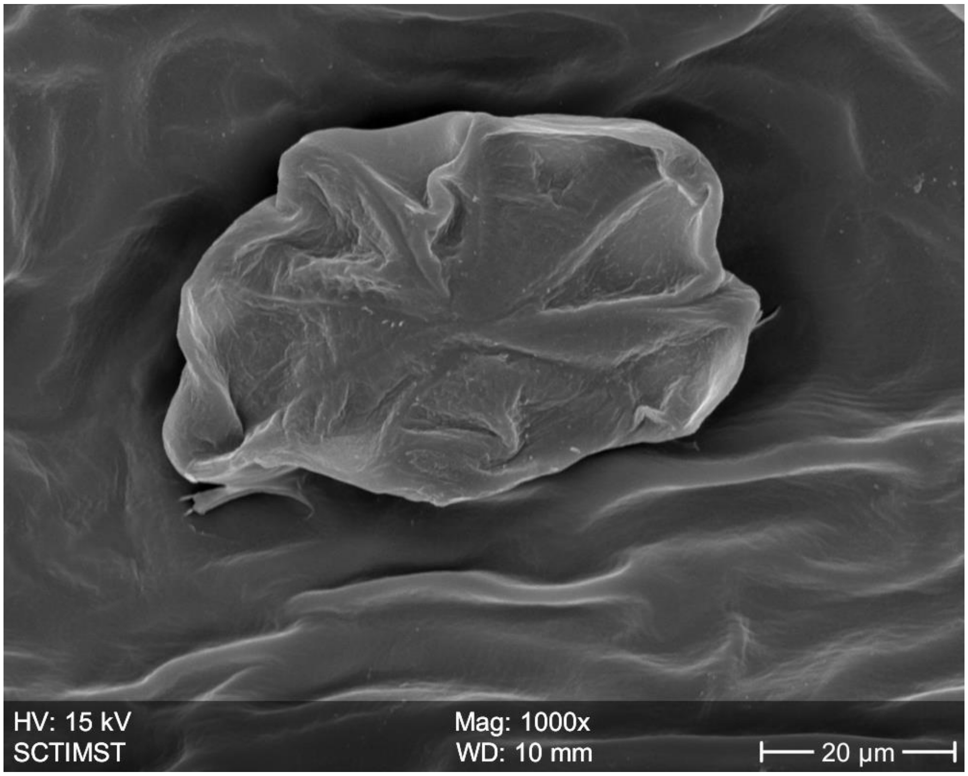

Fig. S13| SEM image of *N. khasiana* pitcher outer side top, glandular trichome (expansion)[1,2].

Table S1. *Nepenthes khasiana* and *Nepenthes* hybrid pitcher weight measurements, before and after cutting them open.

Sl. No.	Species/hybrid (mature-near mature pitchers before lid opening)	Pitcher length (cm)	Lid lateral length (cm)	Pitcher middle, width (cm)	Pitcher lower, width (cm)	Pitcher fluid (ml)	Initial weight (g)	Weight after cutting open (quick re-weighing) (g)	Weight difference (mg)
Nepenthes khasiana									
1.	*N. khasiana*	14.0	3.7	1.8	2.4	6.2	12.3967	12.3945	2.2
2.	*N. khasiana*	12.5	2.6	1.5	1.9	3.2	6.8854	6.8841	1.3
3.	*N. khasiana*	11.5	3.4	1.2	1.2	1.0	4.4841	4.4821	2.0
4.	*N. khasiana*	11.4	2.3	1.2	1.5	2.0	4.4908	4.4897	1.1
5.	*N. khasiana*	11.6	2.7	1.5	1.7	1.8	4.5250	4.5236	1.4
6.	*N. khasiana*	11.7	2.5	1.4	1.4	2.0	4.7125	4.7113	1.2
7.	*N. khasiana*	11.0	2.4	1.1	1.2	1.6	3.8831	3.8815	1.6
8.	*N. khasiana*	11.5	2.7	1.5	1.6	2.0	5.0920	5.0908	1.2
9.	*N. khasiana*	8.5	2.2	1.0	1.1	1.0	2.6939	2.6921	1.8
10.	*N. khasiana*	10.0	2.1	1.0	1.1	0.4	1.9514	1.9499	1.5

11.	*N. khasiana*	17.2	4.5	2.0	2.5	5.0	12.6552	12.6504	4.8
12.	*N. khasiana*	12.3	2.9	2.4	2.6	2.8	7.5910	7.5882	2.8
13.	*N. khasiana*	13.7	3.2	2.2	2.3	2.3	6.6739	6.6715	2.4
14.	*N. khasiana*	9.7	2.1	1.3	1.2	0.7	2.1711	2.1684	2.7
15.	*N. khasiana*	7.2	2.0	1.1	1.4	1.1	3.0467	3.0452	1.5
16.	*N. khasiana*	8.3	2.0	1.1	1.2	0.6	2.6235	2.6225	1.0
17.	*N. khasiana*	13.0	2.7	2.5	2.0	2.1	5.5132	5.5106	2.6
18.	*N. khasiana*	11.2	2.9	2.4	2.3	2.2	4.9334	4.9314	2.0
19.	*N. khasiana*	13.2	3.4	2.7	2.4	2.7	6.8130	6.8087	4.3
20.	*N. khasiana*	9.2	1.8	1.1	1.3	1.1	3.3640	3.3629	1.1
21.	*N. khasiana*	8.6	1.8	1.2	1.2	1.0	2.6526	2.6515	1.1
22.	*N. khasiana*	6.2	1.4	0.9	1.1	0.4	1.0591	1.0583	0.8
23.	*N. khasiana*	13.8	2.8	1.3	1.6	1.4	4.8401	4.8348	5.3
24.	*N. khasiana*	14.3	3.3	1.2	1.8	2.6	5.9970	5.9935	3.5
25.	*N. khasiana*	11.3	2.5	1.2	1.5	1.2	4.6888	4.6863	2.5
26.	*N. khasiana*	9.5	2.3	1.2	1.4	1.6	3.3472	3.3455	1.7

27.	*N. khasiana*	15.6	3.0	1.6	2.5	4.0	8.9423	8.9405	1.8
28.	*N. khasiana*	15.1	3.0	1.7	2.2	4.0	8.7290	8.7267	2.3
29.	*N. khasiana*	18.6	4.2	2.0	2.4	5.5	13.1310	13.1285	2.5
30.	*N. khasiana*	18.7	4.2	1.9	2.5	6.0	14.1763	14.1723	4.0
31.	*N. khasiana*	12.8	2.7	1.8	1.8	4.0	7.8101	7.8079	2.2
32.	*N. khasiana*	16.1	3.8	2.1	2.3	4.5	10.9142	10.9086	5.6
33.	*N. khasiana*	13.2	3.1	2.7	2.8	5.2	9.0007	8.9990	1.7
34.	*N. khasiana*	14.3	3.4	2.5	2.6	4.2	9.2075	9.2065	1.0
35.	*N. khasiana*	16.8	3.8	2.1	2.4	5.0	12.1587	12.1551	3.6
36.	*N. khasiana*	15.8	3.7	2.4	2.4	4.0	10.5566	10.5525	4.1
37.	*N. khasiana*	13.0	3.2	2.1	2.3	3.4	7.3150	7.3133	1.7
38.	*N. khasiana*	14.0	3.4	2.5	2.3	5.1	9.9722	9.9704	1.8
39.	*N. khasiana*	14.2	3.0	2.1	2.5	4.3	9.4068	9.4048	2.0
40.	*N. khasiana*	12.4	3.2	2.1	2.2	3.3	6.6287	6.6273	1.4
41.	*N. khasiana*	12.7	3.1	2.3	2.2	3.1	6.4174	6.4156	1.8
42.	*N. khasiana*	15.3	3.1	2.1	2.3	3.7	8.6193	8.6174	1.9

43.	*N. khasiana*	20.1	4.9	2.8	3.6	11.0	24.4500	24.4415	8.5
44.	*N. khasiana*	18.2	4.5	3.7	3.4	9.7	18.6464	18.6404	6.0
45.	*N. khasiana*	13.0	4.1	3.4	3.6	6.3	13.1345	13.1314	3.1
Mean ± s.d.		12.94 ± 3.11 (n = 45)	3.01 ± 0.79 (n = 45)	1.84 ± 0.67 (n = 45)	2.03 ± 0.66 (n = 45)	3.25 ± 2.29 (n = 45)	7.5178 ± 4.6290 (n = 45)	7.5153 ± 4.6279 (n = 45)	2.50 ± 1.58 (n = 45)
Nepenthes hybrids									
1.	*Nepenthes* hybrid 1	11.5	4.4	4.0	3.6	6.8	14.8815	14.8760	5.5
2.	*Nepenthes* hybrid 1	14.0	5.5	2.9	3.4	6.5	14.1581	14.1528	5.3
3.	*Nepenthes* hybrid 1	11.0	4.6	1.1	2.1	5.0	12.6965	12.6916	4.9
4.	*Nepenthes* hybrid 1	14.5	6.8	2.9	3.8	12.0	23.4216	23.4168	4.8
Mean ± s.d.		12.75 ± 1.76 (n = 4)	5.33 ± 1.09 (n = 4)	2.73 ± 1.20 (n = 4)	3.23 ± 0.77 (n = 4)	7.58 ± 3.05 (n = 4)	16.2894 ± 4.8409 (n = 4)	16.2843 ± 4.8410 (n = 4)	5.13 ± 0.33 (n = 4)
1.	*Nepenthes* hybrid 2	8.0	3.5	1.9	2.6	9.8	15.3419	15.3377	4.2
2.	*Nepenthes* hybrid 2	11.3	3.9	2.3	2.8	7.8	13.0338	13.0306	3.2

3.	*Nepenthes* hybrid 2	12.7	6.0	3.1	4.3	11.7	19.8237	19.8203	3.4
4.	*Nepenthes* hybrid 2	9.8	4.5	1.6	2.5	7.0	12.2218	12.2191	2.7
Mean ± s.d.		10.45 ± 2.02 (n = 4)	4.48 ± 1.10 (n = 4)	2.23 ± 0.65 (n = 4)	3.05 ± 0.84 (n = 4)	9.08 ± 2.11 (n = 4)	15.1053 ± 3.4120 (n = 4)	15.1019 ± 3.4117 (n = 4)	3.38 ± 0.62 (n = 4)
1.	*Nepenthes* hybrid 3	13.1	3.9	2.7	2.5	3.3	12.0819	12.0800	1.9
Mean		13.10 (n = 1)	3.90 (n = 1)	2.70 (n = 1)	2.50 (n = 1)	3.30 (n = 1)	12.0819 (n = 1)	12.0800 (n = 1)	1.9
1.	*Nepenthes* hybrid 4	5.5	1.1	1.2	1.6	0.7	1.3117	1.3108	0.9
2.	*Nepenthes* hybrid 4	6.5	1.2	1.1	1.6	2.0	3.0949	3.0938	1.1
Mean		6.00 (n = 2)	1.15 (n = 2)	1.15 (n = 2)	1.60 (n = 2)	1.35 (n = 2)	2.2033 (n = 2)	2.2023 (n = 2)	1.0 (n = 2)
1.	*Nepenthes* hybrid 5	4.2	1.7	0.9	1.1	0.9	2.0146	2.0139	0.7
Mean		4.20 (n = 1)	1.70 (n = 1)	0.90 (n = 1)	1.10 (n = 1)	0.90 (n = 1)	2.0146 (n = 1)	2.0139 (n = 1)	0.7 (n = 1)
1.	*Nepenthes* hybrid 6	4.2	1.5	0.9	1.2	0.4	1.1238	1.1226	1.2

Mean	4.20<br>(n = 1)	1.50<br>(n = 1)	0.90<br>(n = 1)	1.20<br>(n = 1)	0.40<br>(n = 1)	1.1238<br>(n = 1)	1.1226<br>(n = 1)	1.2<br>(n = 1)

Table S2. Growth parameters of *Nepenthes khasiana* cut and uncut pitchers.

	Test (cut) pitchers					Control (uncut) normal pitchers				
Sl. No.	Initial pitcher length (cm) (6-8 cm)	Pitcher length at lid opening (cm)	Growth (in cm)	No. of days of growth	Growth (cm per day)	Initial pitcher length (cm) (6-8 cm)	Pitcher length at lid opening (cm)	Growth (in cm)	No. of days of growth	Growth (cm per day)
1.	6.1	14.6	8.5	13	0.65	5.4	16.5	11.1	13	0.85
2.	6.0	15.4	9.4	13	0.72	6.4	14.8	8.4	10	0.84
3.	7.0	12.7	5.7	12	0.48	5.6	11.6	6.0	12	0.50
4.	6.3	12.4	6.1	11	0.55	5.8	14.5	8.7	12	0.73
5.	8.0	15.0	6.9	10	0.69	6.1	12.9	6.8	10	0.68
6.	7.0	13.9	6.9	13	0.53	5.9	16.6	10.7	14	0.76
7.	7.0	13.1	6.1	11	0.55	7.0	16.8	9.8	11	0.89
8.	7.1	15.8	8.7	14	0.62	8.0	16.4	8.4	10	0.84
9.	8.0	15.3	7.3	14	0.52	5.5	13.9	8.4	13	0.64

10.	6.3	12.1	5.8	10	0.58	6.8	18.5	11.7	14	0.83
11.	7.6	14.0	6.4	10	0.64	5.9	15.2	9.3	14	0.66
12.	6.0	17.0	11.0	17	0.65	5.8	13.8	8.0	16	0.50
13.	6.4	12.9	6.5	13	0.50	6.0	18.7	12.7	16	0.79
14.	7.5	14.2	6.7	12	0.56	7.0	14.9	7.9	11	0.66
15.	6.5	10.3	3.8	12	0.32	6.9	16.6	9.7	14	0.69
16.	6.9	11.2	4.3	12	0.36	7.7	12.7	5.0	7	0.71
17.	7.5	16.4	8.9	12	0.74	6.3	10.6	4.3	13	0.33
18.	8.0	14.3	6.2	16	0.39	6.0	9.1	3.1	6	0.51
19.	7.4	13.8	6.4	16	0.40	6.7	15.9	9.2	12	0.76
20.	5.7	13.7	8.0	15	0.53	7.0	13.8	6.8	10	0.68
21.	7.6	18.1	10.5	12	0.88	7.2	15.8	8.6	16	0.54
22.	6.0	13.4	7.4	14	0.53	6.1	12.8	6.7	11	0.6
23.	7.9	15.1	7.2	13	0.55	5.3	12.1	6.8	15	0.45
24.	7.6	12.2	4.6	11	0.42	5.0	13.9	8.9	15	0.56
25.	5.6	11.4	5.8	11	0.53	7.6	17.3	9.7	11	0.88

26.	7.4	13.4	6.0	10	0.60	5.7	13.9	8.2	12	0.68
27.	6.9	13.6	6.7	9	0.74	6.3	12.1	5.8	13	0.45
28.	6.8	15.8	9.0	10	0.90	5.3	11.4	6.1	11	0.55
29.	6.9	11.5	4.6	8	0.58	6.1	11.2	5.1	11	0.46
30.	7.4	11.6	4.2	6	0.70	7.2	20.1	12.9	12	1.08
31.	6.5	13.7	7.2	11	0.65	6.2	12.5	6.3	7	0.90
32.	7.2	16.2	9.0	11	0.82	6.1	15.9	9.8	10	0.98
33.	7.3	13.4	6.1	10	0.61	6.1	14.3	8.2	10	0.82
34.	6.2	15.6	9.4	12	0.78	6.8	12.1	5.3	7	0.76
35.	7.2	13.8	6.6	10	0.66	6.3	14.8	8.5	9	0.94
36.	6.1	10.0	3.9	7	0.56	5.6	14.0	8.4	10	0.84
37.	7.5	11.2	3.7	8	0.46	7.2	16.6	9.4	9	1.04
38.	7.0	11.7	4.7	8	0.59	5.9	16.1	10.2	12	0.85
39.	5.6	14.1	8.5	11	0.77	5.2	13.6	8.4	10	0.84
40.	6.8	11.6	4.8	8	0.60	6.1	10.2	4.1	7	0.59
41.	7.5	14.9	7.4	11	0.68	6.0	12.8	6.8	9	0.76

42.	6.6	13.1	6.5	10	0.65	5.6	13.6	8	12	0.67
43.	6.3	18.0	11.7	12	0.98	6.8	17.0	10.2	11	0.93
44.	5.5	15.3	9.8	16	0.61	6.2	14.5	8.3	16	0.52
45.	7.4	10.5	3.1	7	0.44	7.1	10.5	3.4	7	0.49
Mean ± s.d.	6.87 ± 0.70 (n = 45)	13.72 ± 1.96 (n = 45)	6.84 ± 2.03 (n = 45)	11.38 ± 2.53 (n = 45)	0.61 ± 0.14 (n = 45)	6.28 ± 0.71 (n = 45)	14.29 ± 2.42 (n = 45)	8.00 ± 2.28 (n = 45)	11.36 ± 2.66 (n = 45)	0.71 ± 0.17 (n = 45)

References

1. Subramanyam, K. & Narayana, L. L. A contribution to the floral anatomy of *Nepenthes khasiana* Hook F. *Proc. Indian Acad. Sci. B.* **73**, 124-131 (1971).
2. Gaume, L., Gorb, S. & Rowe, N. Functional epidermal surfaces in the trapping efficiency of *Nepenthes alata* pitchers. *New Phytol.* **156**, 479-489 (2002).
